# Reaction mechanism of atomic layer deposition of zirconium oxide using zirconium precursors bearing amino ligands and water

**DOI:** 10.3389/fchem.2022.1035902

**Published:** 2022-11-04

**Authors:** Rui Xu, Zhongchao Zhou, Jing Li, Xu Zhang, Yuanyuan Zhu, Hongping Xiao, Lina Xu, Yihong Ding, Aidong Li, Guoyong Fang

**Affiliations:** ^1^ Key Laboratory of Carbon Materials of Zhejiang Province, College of Chemistry and Materials Engineering, Wenzhou University, Wenzhou, China; ^2^ National Laboratory of Solid State Microstructures, College of Engineering and Applied Sciences, Nanjing University, Nanjing, China

**Keywords:** zirconium oxide, atomic layer deposition, reaction mechanism, tetrakis(dimethylamino)zirconium, density functional theory

## Abstract

As a unique nanofabrication technology, atomic layer deposition (ALD) has been widely used for the preparation of various materials in the fields of microelectronics, energy and catalysis. As a high-κ gate dielectric to replace SiO_2_, zirconium oxide (ZrO_2_) has been prepared through the ALD method for microelectronic devices. In this work, through density functional theory calculations, the possible reaction pathways of ZrO_2_ ALD using tetrakis(dimethylamino)zirconium (TDMAZ) and water as the precursors were explored. The whole ZrO_2_ ALD reaction could be divided into two sequential reactions, TDMAZ and H_2_O reactions. In the TDMAZ reaction on the hydroxylated surface, the dimethylamino group of TDMAZ could be directly eliminated by substitution and ligand exchange reactions with the hydroxyl group on the surface to form dimethylamine (HN(CH_3_)_2_). In the H_2_O reaction with the aminated surface, the reaction process is much more complex than the TDMAZ reaction. These reactions mainly include ligand exchange reactions between the dimethylamino group of TDMAZ and H_2_O and coupling reactions for the formation of the bridged products and the by-product of H_2_O or HN(CH_3_)_2_. These insights into surface reaction mechanism of ZrO_2_ ALD can provide theoretical guidance for the precursor design and improving ALD preparation of other oxides and zirconium compounds, which are based ALD reaction mechanism.

## 1 Introduction

As an excellent nanofabrication technology, atomic layer deposition (ALD) can prepare large-area, uniform and conformal thin films at the atomic level (Klaus et al., 1997; Ritala et al., 2000; Hausmann et al., 2002; Chen et al., 2011). Meanwhile, the compositions and structures of thin films can also be controlled through varying the number of ALD cycles and precursors. ALD is a type of chemical vapor deposition (CVD) technique, namely, atomic layer chemical vapor deposition (ALCVD). It can divide the whole CVD reaction into several separate surface reactions. It can have the features of self-limitation and take full advantage of the gas-solid surface reactions. Currently, ALD has been widely used in the fields of microelectronics, nanotechnology, catalysis and energy, etc. (Zaera, 2008; Rolison et al., 2009; Marichy et al., 2012; O'Neill et al., 2015; Palmstrom et al., 2015; Asundi et al., 2019).

As the core of microelectronics technology, the development of large-scale integrated circuits obeys Moore’s law. Since the beginning of the 21st century, the thickness of SiO_2_ gate dielectrics in MOSFET devices has continuously decreased. However, the tunneling effect of electrons leads to significant leakage and power consumption and seriously affects the stability and reliability of MOSFET devices. Currently, using high-κ gate dielectrics to replace SiO_2_ is an effective method for solving the problem. Because of the high dielectric constant and thermodynamic stability, zirconium oxide (ZrO_2_) has been used as gate dielectrics *via* the ALD method for MOSFET devices (Gaskell et al., 2007; Dezelah IV et al., 2008; Kaipio et al., 2014; Jung et al., 2015; Kanomata et al., 2016; Mahuli et al., 2021; Xu et al., 2021).

In general, the prerequisite and key to the success of ALD technology require suitable precursors. For ZrO_2_ ALD, the zirconium precursors include these linked by alkyl, halide and alkoxy ligands, such as Zr(Cp)_2_, ZrCl_4_ and Zr(OEt_2_)_4_ (Williams et al., 2002; Yoshii et al., 2002; Niinistö et al., 2005; Knapas and Ritala, 2008). Subsequently, the zirconium precursor bearing amino ligands is also a candidate for ZrO_2_ ALD. Because of good volatility, thermal stability and high reactivity, tetrakis(dimethylamino)zirconium (TDMAZ, Zr(NMe_2_)_4_) has been studied (Provine et al., 2016). Different precursors have different effects on the overall ALD reaction. Experimentally, thermal ALD of ZrO_2_ can be performed using Zr(NMe_2_)_4_ as the zirconium source and H_2_O as the oxygen source. It can be written as two separate reactions as follows:

$$\left(A\right)\;{\text{ZrO}}_{2}{-\text{OH}}^{*}+\text{Zr}{\left({\text{NMe}}_{2}\right)}_{4}\to {\text{ZrO}}_{2}{-O-\text{ZrNMe}}_{2}^{*}{+\text{HNMe}}_{2}$$

$$\left(B\right)\;{\text{ZrO}}_{2}{-O-\text{ZrNMe}}_{2}^{*}+H_{2}O\to {\text{ZrO}}_{2}{-O-\text{ZrOH}}^{*}{+\text{HNMe}}_{2}$$

where an asterisk designates a surface species.

To obtain more insight into the ALD reaction mechanism of various materials, many theoretical calculations have been performed (Elliott, 2012; Hu et al., 2015; Elliott et al., 2016). These works include density functional theory (DFT) calculations, molecular dynamics and Monte Carlo simulations. For example, the ALD mechanism of oxides and nitrides, such as SiO_2_, Si_3_N_4_, Al_2_O_3_, TiO_2_, ZrO_2_ and HfO_2_, have been widely explored (Mukhopadhyay et al., 2008; Ren et al., 2008; Han et al., 2012; Huang et al., 2013; Huang et al., 2014). To date, only a few mechanisms of ZrO_2_ ALD using precursors with halide and alkyl ligands, such as ZrCl_4_ and ZrCp_2_Me_2_, have been studied (Brodskii et al., 2002; Jeloaica et al., 2003; Jeloaica et al., 2005; Ren et al., 2011; Zhou et al., 2013). However, the investigation of more effective zirconium precursors bearing amino ligands and their roles and reaction mechanism for ZrO_2_ ALD is still lacking.

**FIGURE 1:**
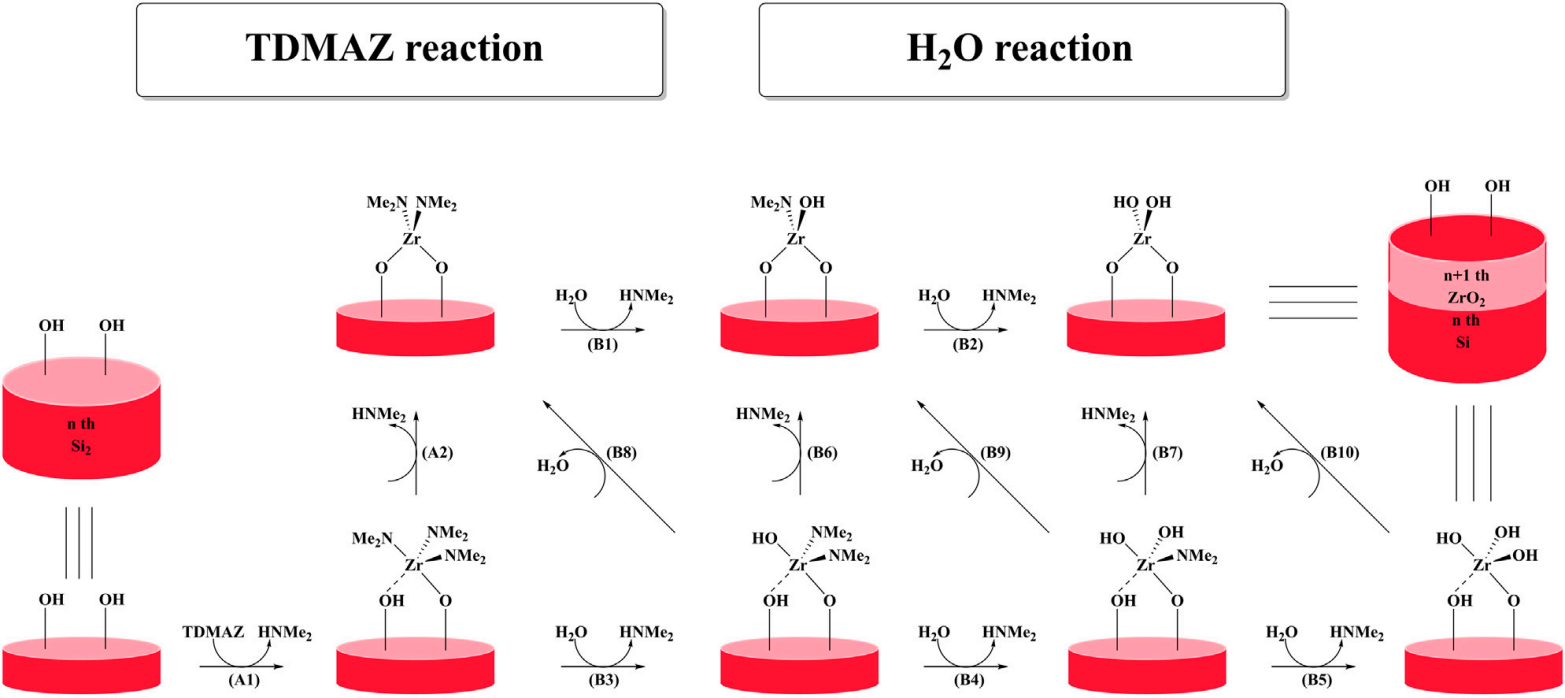
Possible pathways of ZrO_2_ ALD using TDMAZ and H_2_O as precursors.

Herein, we investigated the reactions of Zr(NMe_2_)_4_ and H_2_O on surfaces to gain more insight into the reaction mechanism of ZrO_2_ ALD using DFT. The whole reaction of ZrO_2_ ALD includes the TDMAZ half-reaction (**A1** and **A2**) and H_2_O half-reaction (**B1**–**B10**), as shown in Figure 1. The results show that both TDMAZ and H_2_O can react with the hydroxyl and amino groups on the surface. These insights into the reaction mechanism of ZrO_2_ ALD can improve precursor design and ALD growth for other oxides and zirconium compounds and boost the further development of ALD chemistry.

## 2 Computational details

**FIGURE 2:**
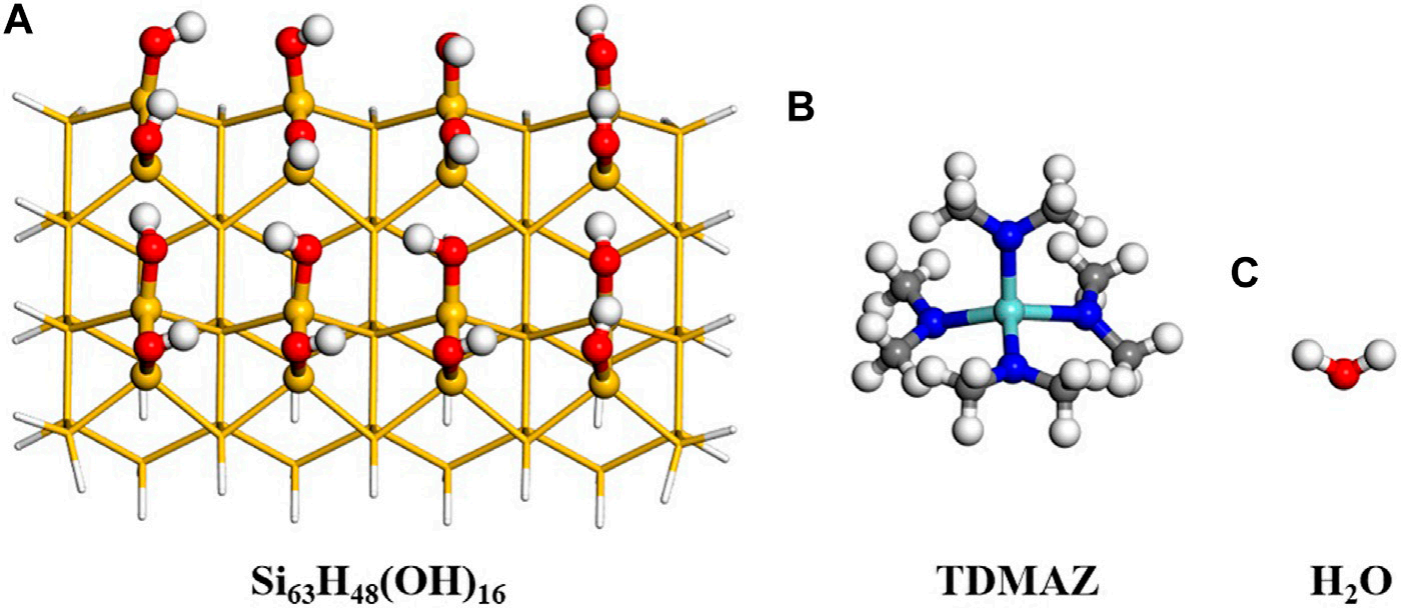
Surface model for Si_63_H_48_(OH)_16_, TDMAZ and H_2_O. The yellow, red, white, blue and light blue balls represent Si, O, H, N and Zr atoms, respectively.

To model the surface reaction of ZrO_2_ ALD, the cluster model Si_63_H_48_(OH)_16_ was adopted and shown in Figure 2. In general, silicon is used as a substrate material in microelectronic devices. The cluster model is based on the hydroxylated Si(001) surface with four layers of silicon atoms and sixteen hydroxyl (–OH) groups. Our previous and other works both proved that when the size of the surface is larger than the size of precursor molecules, the cluster and slab models can give similar results (Mukhopadhyay et al., 2008; Fang and Ma, 2013). The suspended bonds of the model are saturated with H atoms. To model the surface, the three layers of Si atoms at the bottom are fixed and sixteen Si atoms and hydroxyl groups on the surface are relaxed. The precursors include TDMAZ and H_2_O as shown in Figure 2.

All species in ZrO_2_ ALD reactions were optimized using DFT with the M06-2X functional. The M06-2X functional is one of the most suitable functionals to describe the interactions between the precursors and the surface (Zhao and Truhlar, 2008a; Zhao and Truhlar, 2008b). This functional was also tested using the precursor reaction on the hydroxylated surface in previous work (Fang and Ma, 2013). By comparing different density functionals (M06-2X, PBEPBE and B3LYP) with the MP2 method, it was found that the M06-2X functional is appropriate for ALD surface reactions (Fang and Ma, 2013). Meanwhile, the dispersion correction for weak interactions was performed using Grimme dispersion method with the original D3 damping function (GD3) (Grimme et al., 2010; Grimme et al., 2011). To balance the computational accuracy and cost, the 6-311G(d,p) basis set was used for the relaxed atoms and adsorbates on the surface and the LANL2DZ basis set was used for the Zr atom. Other atoms of the substrate at the bottom were described using the 6-31G basis set. All stationary points and transition states were verified using frequency and intrinsic reaction coordinate (IRC) calculations. The Gibbs free energies were also calculated from the partition functions, as well as the enthalpy and entropy terms at different temperatures (298.15 and 473.15 K) and pressures (1 atm and 0.2 Torr) (Baletto and Ferrando, 2005; Levine, 2008; Li and Truhlar, 2014). Notably, the precursor molecules in the gas phase have three motions of rotation, translation, and vibration. When the precursors are adsorbed on the surface, the rotation and translation motions are lost and new vibrations are produced. In other words, the entropy of the surface has no contribution from translation and rotation, and only has the contribution from the vibrations (Zhou et al., 2022). All optimization, frequency and IRC calculations were performed with the Gaussian 09 program (Frisch et al., 2013). The corresponding thermodynamic properties were calculated by shermo program (Lu and Chen, 2021).

## 3 Results and discussion

### 3.1 Tetrakis(dimethylamino)zirconium reaction on the hydroxylated surface

#### 3.1.1 The elimination of the first amino ligand of tetrakis(dimethylamino)zirconium *via* the A1 reaction

**FIGURE 3:**
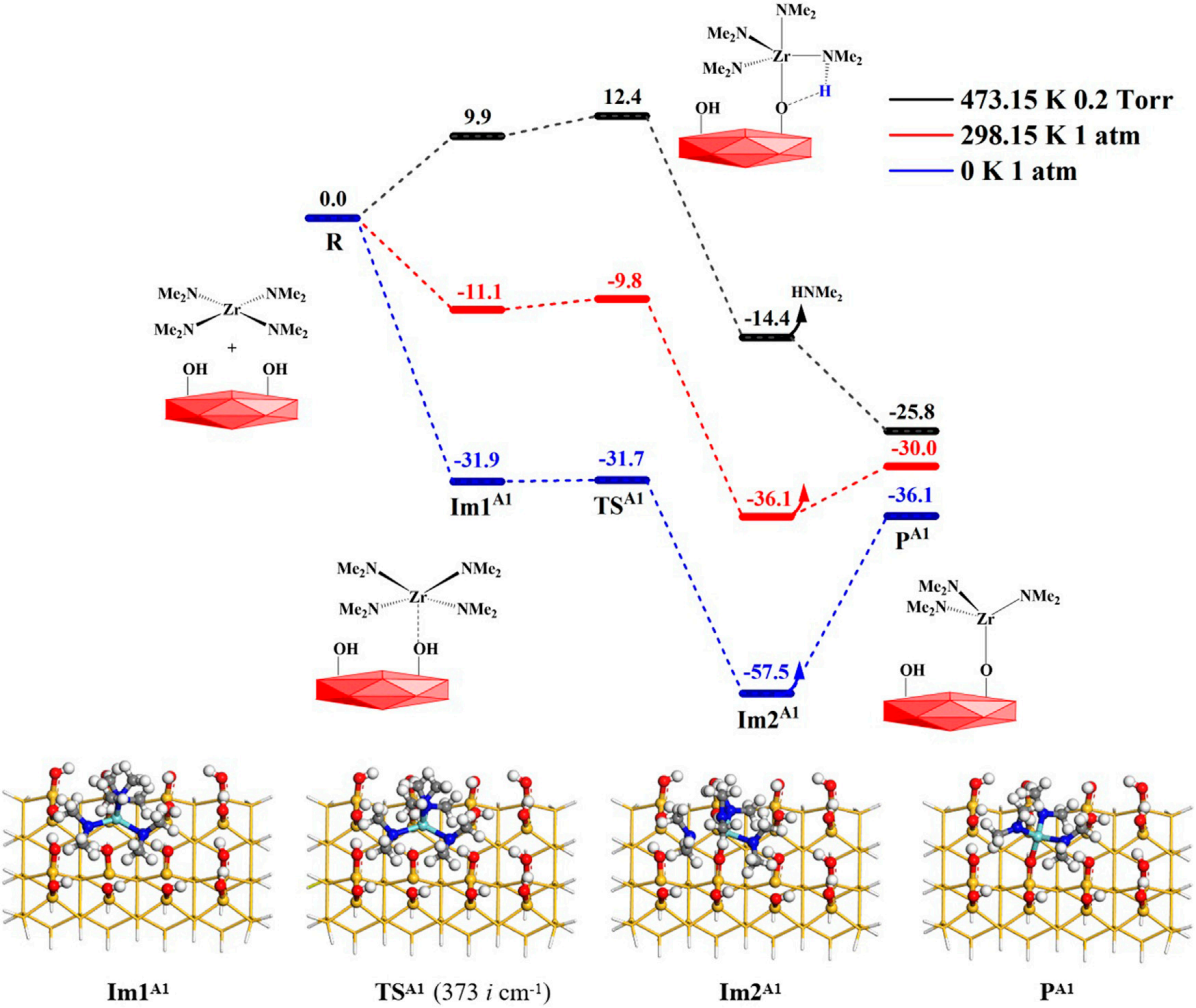
Gibbs free energy profiles (Δ*G*, kcal/mol) of the **A1** section of the TDMAZ reaction on the hydroxylated surface.

When the precursor tetrakis(dimethylammonium)zirconium (TDMAZ) approaches the hydroxylated surface, it can undergo two steps (**A1** and **A2**) of the substitution and elimination of amino ligands. The Gibbs free energy profiles of the elimination reaction (**A1**) of the first amino ligand are shown in Figure 3. First, TDMAZ can be adsorbed on the hydroxylated surface to form intermediate **Im1** ^**A1**^ with the adsorption energy (***E***_**ads**_) of 31.9 kcal/mol. Then, it can undergo a four-membered ring (4MR) transition state (**TS** ^**A1**^) with very low activation energy (***E***_**a**_) and the imaginary frequency of 373 cm^−1^. In **TS** ^**A1**^, the Zr atom of the precursor can attack the O atom of the hydroxyl group on the surface. At the same time, the H atom of the hydroxyl group can be transferred to the N atom of the amino ligand of the precursor. Later, the intermediates **Im2** ^**A1**^ and dimethylamine (HNMe_2_) can be generated. Eventually, the product **P** ^**A1**^ (–OZr(NMe_2_)_3_) can be formed and the HNMe_2_ molecule can be released from the surface, in which the desorption energy (***E***_**des**_) of dimethylamine is 21.4 kcal/mol. The bond length changes at the reaction center are listed in Table 1. In the reaction process, the lengths of the O–H and Zr–N bonds increase from 0.980 to 2.072 Å in **Im1** ^**A1**^ to 2.536 and 2.368 Å in **Im2** ^**A1**^, respectively. The lengths of the Zr–O and H–N bonds decrease from 2.481 to 2.119 Å in **Im1** ^**A1**^ to 2.076 and 1.019 Å in **Im2** ^**A1**^, respectively. All these indicate that O–H and Zr–N bonds are broken and Zr–O and H–N bonds are formed.

**TABLE 1:** Bond lengths (Å) at the reaction center in the **A1** section of the TDMAZ reaction on the hydroxylated surface.

Species	O–H	Zr–N	Zr–O	H–N
**Im1** ^**A1**^	0.980	2.072	2.481	2.119
**TS** ^**A1**^	1.088	2.164	2.394	1.506
**Im2** ^**A1**^	2.536	2.368	2.076	1.019

In general, the temperatures and pressures have a certain effect on the ALD surface reaction. As shown in Figure 3, at 298.15 K and 1 atm, the Gibbs activation energy (*G*_a_) of the **A1** reaction is very low at only 1.3 kcal/mol, which indicates that the reaction can easily occur at room temperature. The desorption of dimethylamine is also easy and requires a low desorption energy of about 6.0 kcal/mol. As a whole, the **A1** reaction is exoergic by 30.0 kcal/mol. At 473.15 K and 0.2 Torr, the free energies of the intermediates, **Im1** ^**A1**^ and **Im2** ^**A1**^, and transition state **TS** ^**A1**^ further increase because of temperature and entropy effects. The Gibbs activation energy of the **A1** reaction increases to 12.4 kcal/mol. All of these indicate that the **A1** reaction of TDMAZ on the hydroxylated surface is thermodynamically and kinetically favorable at the experimental condition of 473.15 K and 0.2 Torr.

#### 3.1.2 The elimination of the second amino ligand of tetrakis(dimethylamino)zirconium *via* the A2 reaction

**FIGURE 4:**
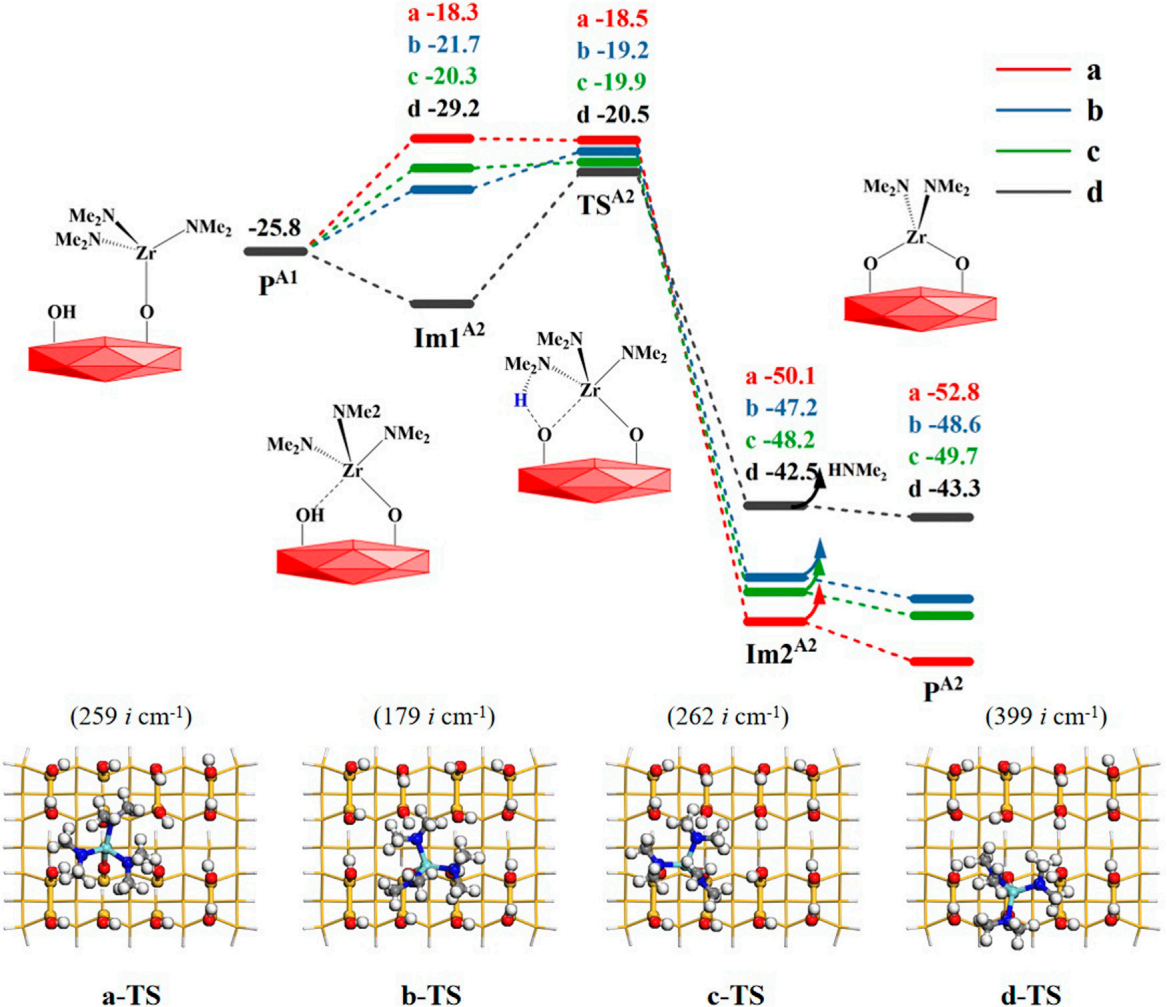
Gibbs free energy profiles (Δ*G*, kcal/mol) of the **A2** section of the TDMAZ reaction with the hydroxylated surface at 473.15 K and 0.2 Torr. Symbols a, b, c and d represent four different directions of –OZr(NMe_2_)_3_ with adjacent hydroxyl groups.

The product **P** ^**A1**^ (–OZr(NMe_2_)_3_) of the **A1** section can react further with adjacent hydroxyl groups. In the **A2** step, the elimination reaction of the second amino ligand of TDMAZ, there are four available hydroxyl groups on the hydroxylated surface in different directions, **a**, **b**, **c** and **d**, to form **Im1** ^**A2**^, shown in Figure 4. Similar to the **A1** step, the **A2** reaction pathway also goes through a 4MR transition state **TS** ^**A2**^ to obtain the bridged product **P** ^**A2**^ (–OZr(NMe_2_)_2_O–) and release the small molecule dimethylamine. The corresponding Gibbs activation energies are 7.5, 6.6, 5.9 and 8.7 kcal/mol in the four directions relative to the product **P** ^**A1**^ (–OZr(NMe_2_)_3_), indicating that the elimination of the second amino ligand of TDMAZ can occur easily. This is different from the first amino ligand elimination. In **A1** reaction, the precursor adsorption leads to the reduction of the entropy and requires higher energy barrier of the first amino ligand elimination at certain temperature. In **A2** reaction, the precursor has been anchored on the surface and the change of the entropy has little effect on the reaction barrier. As a result, the **A2** reaction of the second amino ligand is also exoergic by about 20 kcal/mol. The Gibbs free energy of intermediate **Im1** ^**A2−a**^ in the **a** direction is lower than that of transition state **TS** ^**A**2**−a**^, which is caused by the harmonic frequency overestimating the thermal correction of intermediate **Im1** ^**A2−a**^, which is more stable than **TS** ^**A2−a**^ in terms of electronic energy.

According to Table 2, the atomic distances of Zr, O, H and N in the reaction center have similar changes, indicating the breakage of O–H and Zr–N bonds and the formation of Zr–O and H–N bonds. As a whole, all the bond changes in the four directions are similar to each other. Because the product **P** ^**A2−a**^ has the lowest energy and the most stable structure, the product in the **a** direction is used as the initial structure for the next reaction.

**TABLE 2:** Bond lengths (Å) at the reaction center in the **A2** section of the TDMAZ reaction on the hydroxylated surface.

Species	O–H	Zr–N	Zr–O	H–N
**Im1** ^**A2−a**^	0.970	2.119	2.463	2.283
**TS** ^**A2−a**^	1.065	2.236	2.360	1.541
**Im2** ^**A2−a**^	3.102	2.470	2.021	1.019
**Im1** ^**A2−b**^	0.966	2.104	2.435	2.328
**TS** ^**A2−b**^	1.030	2.208	2.406	1.633
**Im2** ^**A2−b**^	3.159	2.465	2.021	1.018
**Im1** ^**A2−c**^	0.968	2.100	2.522	2.230
**TS** ^**A2−c**^	1.042	2.200	2.402	1.597
**Im2** ^**A2−c**^	2.507	2.491	2.038	1.020
**Im1** ^**A2−d**^	0.964	2.072	2.392	2.524
**TS** ^**A2−d**^	1.058	2.212	2.315	1.576
**Im2** ^**A2−d**^	2.637	2.439	2.041	1.021

### 3.2 H_2_O reaction with the aminated surface

#### 3.2.1 H_2_O reaction with the aminated surface *via* the B1 and B2 reactions

After the TDMAZ reaction with the hydroxylated surface, H_2_O can be pumped into the reactor and react with the aminated surface. The H_2_O reaction on the surface is more complex than the TDMAZ reaction with the hydroxylated surface and involves 10 reaction pathways (**B1** to **B10**) to eliminate dimethylamine and water molecules, as shown in Figure 1.

**FIGURE 5:**
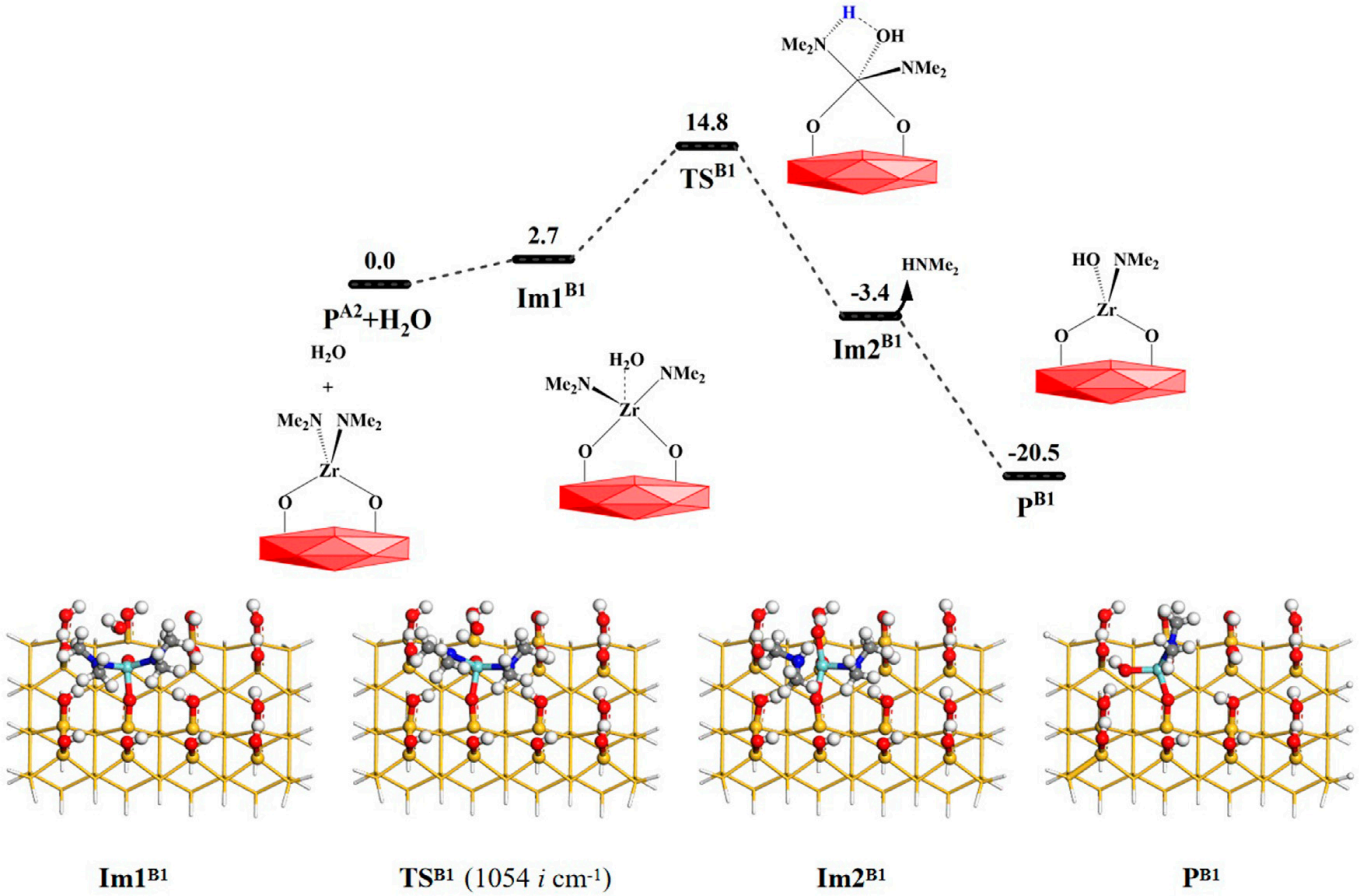
Gibbs free energy (Δ*G*, kcal/mol) profiles of the **B1** section of the H_2_O reaction with the aminated surface.

As shown in Figure 5, a H_2_O molecule and the surface **P** ^**A2**^(–OZr(NMe_2_)_2_O–) can undergo ligand exchange reaction **B1** with the **P** ^**A2**^ (–OZr(NMe_2_)_2_O–) surface. It can sequentially pass through the intermediate **Im1** ^**B**1^, the 4MR transition state **TS** ^**B1**^ and intermediate **Im2** ^**B1**^. Eventually, the HNMe_2_ molecule can be released and the product **P** ^**B1**^ (–OZr(NMe_2_)(OH)O–) can be formed. The **B1** reaction is exoergic by 20.5 kcal/mol. The *G*_a_ of the reaction is 14.8 kcal/mol. From Supplementary Table S1, it can be seen that the distance between H and O atoms increases from 0.962 Å in **Im1** ^**B1**^ to 2.282 Å in **Im2** ^**B1**^, the distance between Zr and N atoms increases from 2.085 to 2.436 Å, whereas the distance between H and N atoms decreases from 2.890 to 1.021 Å, the distance between O and Zr atoms decreases from 2.393 to 2.015 Å. All these indicate that H–O and Zr–N bonds are cleaved and H–N and O–Zr bonds are formed in the **B2** reaction.

**FIGURE 6:**
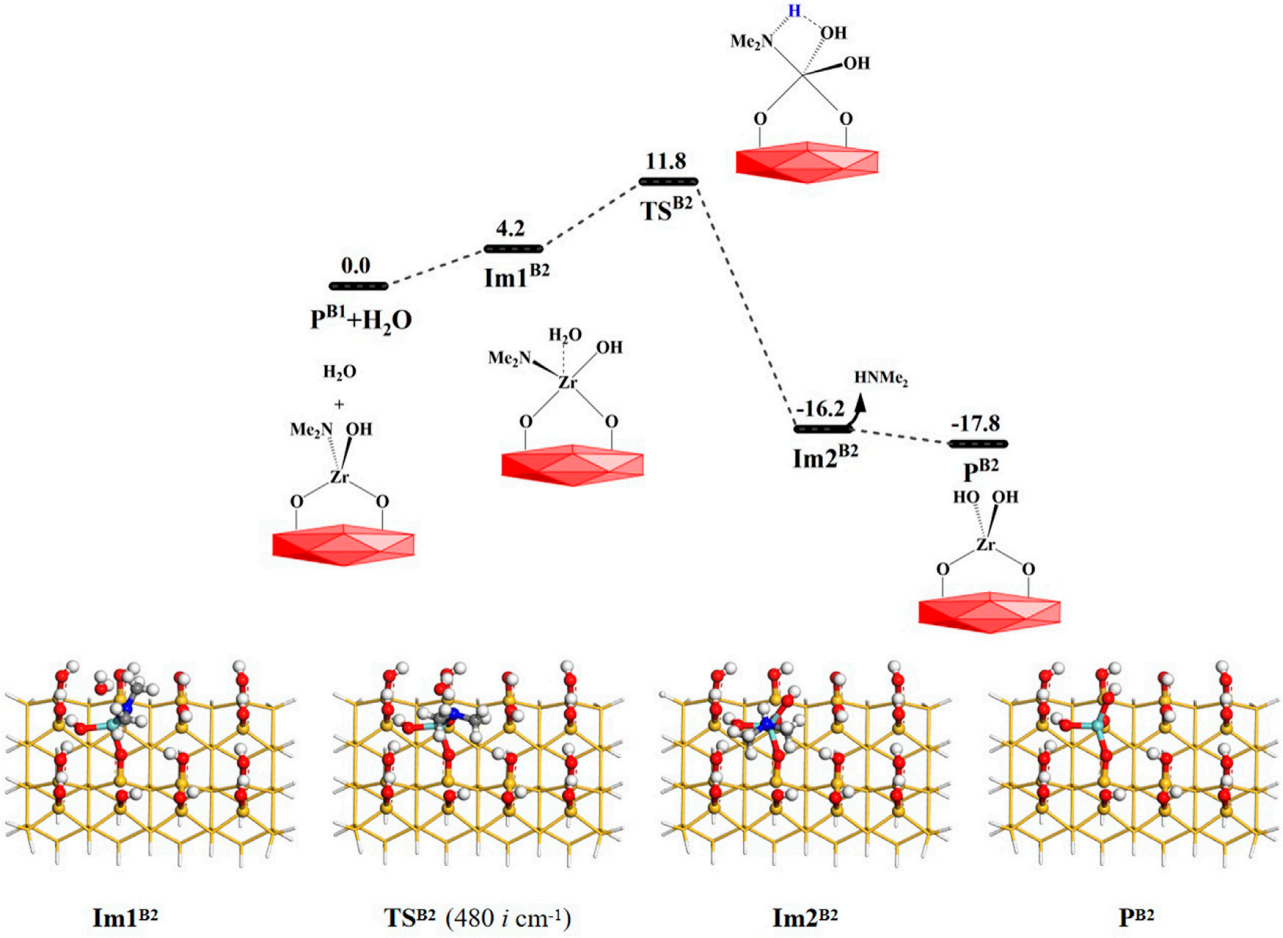
Gibbs free energy (Δ*G*, kcal/mol) profiles of the **B2** section of the H_2_O reaction with the aminated surface.

As shown in Figure 6, the H_2_O molecule can further react with the product **P** ^**B1**^ (–OZr(NMe_2_)(OH)O–). Similar to **B1**, the **B2** reaction is also a ligand exchange reaction between the hydroxyl group and the amino ligand. First, the water molecule can interact with the product **P** ^**B1**^ (–OZr(NMe_2_)(OH)O–) surface to form the intermediate **Im1** ^**B2**^. Subsequently, it can go through a 4MR transition state **TS** ^**B2**^ to form the intermediate **Im2** ^**B2**^. Finally, the HNMe_2_ can be released and the product **P** ^**B2**^ (–OZr(OH)_2_O–) can be obtained. The **B2** reaction is exoergic by 17.8 kcal/mol and the Gibbs activation energy is 11.8 kcal/mol. Supplementary Table S2 lists the changes in the bond lengths at the reaction center of the **B2** reaction. Similar to the **B1** reaction, H–O and Zr–N bonds are gradually broken and H–N and O–Zr bonds are gradually formed during the process of the **B2** reaction. As a whole, **B1** and **B2** reactions are both exoergic and require low energy barriers, indicating that H_2_O and the aminated surface can easily react with each other.

#### 3.2.2 H_2_O reaction with the aminated surface *via* the B3 to B5 reactions

**FIGURE 7:**
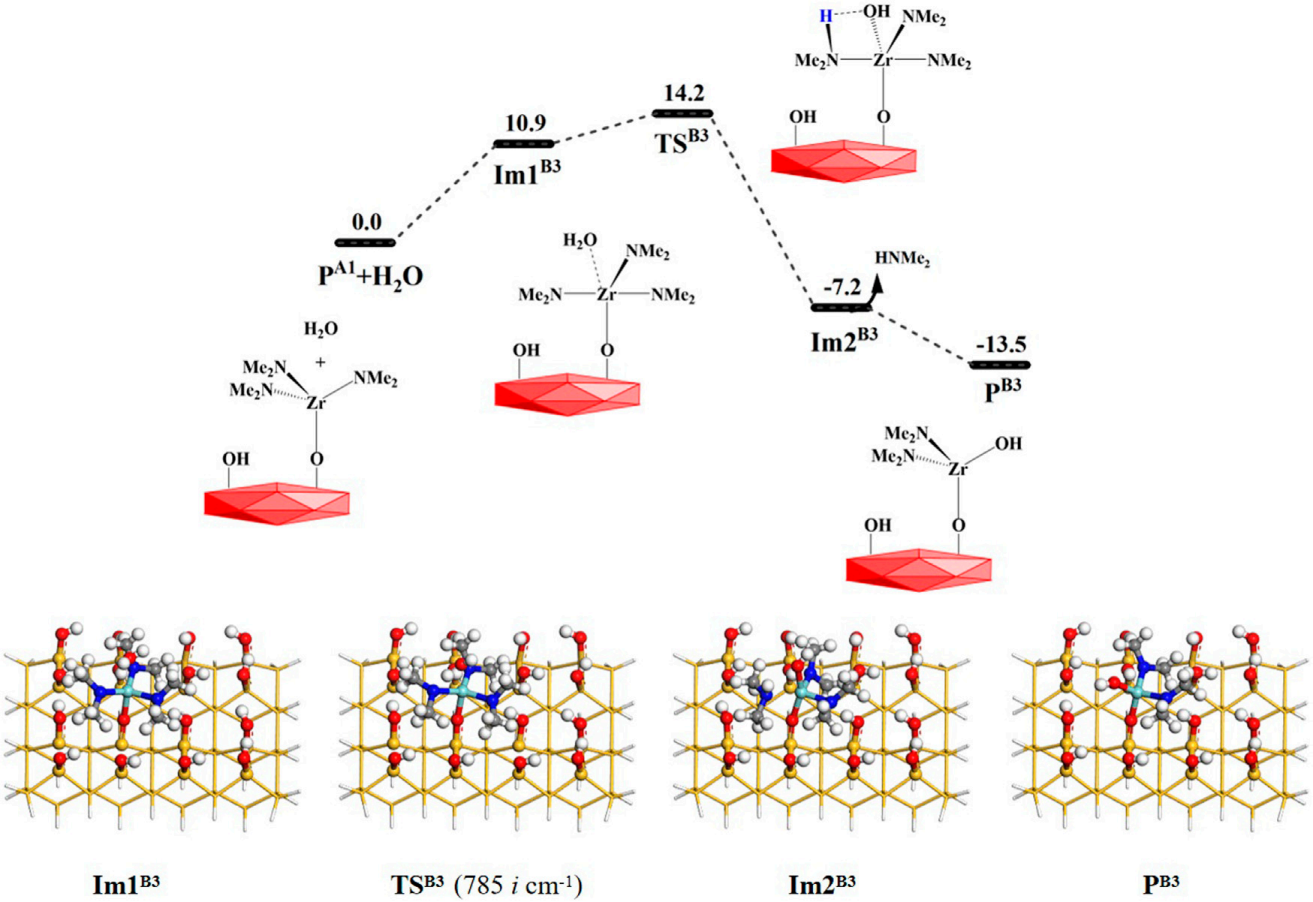
Gibbs free energy (Δ*G*, kcal/mol) profiles of the **B3** section of the H_2_O reaction with the aminated surface.

As mentioned above, the TDMAZ reaction on the hydroxylated surface can form another product **P** ^**A1**^ (–OZr(NMe_2_)_3_), which can also react directly with a water molecule. As shown in Figure 7, H_2_O can react with the product **P** ^**A1**^ (–OZr(NMe_2_)_3_) *via* the **B3** pathway. A water molecule can be adsorbed on the –OZr(NMe_2_)_3_ surface to form an intermediate **Im1** ^**B3**^. It can undergo a 4MR transition state **TS** ^**B3**^ with the Gibbs activation energy of 14.2 kcal/mol and an intermediate **Im2** ^**B3**^. Finally, HNMe_2_ can be released and the product **P** ^**B3**^ (–OZr(NMe_2_)_2_(OH) can be formed, releasing the small molecule. The **B3** reaction is also exoergic by 13.5 kcal/mol. As listed in Supplementary Table S3, the lengths of the H–O and Zr–N bonds gradually increase and the lengths of the H–N and O–Zr bonds gradually decrease. All these indicate that the H–O and Zr–N bonds at the reaction center are broken and the H–N and O–Zr bonds are formed during the **B3** reaction.

**FIGURE 8:**
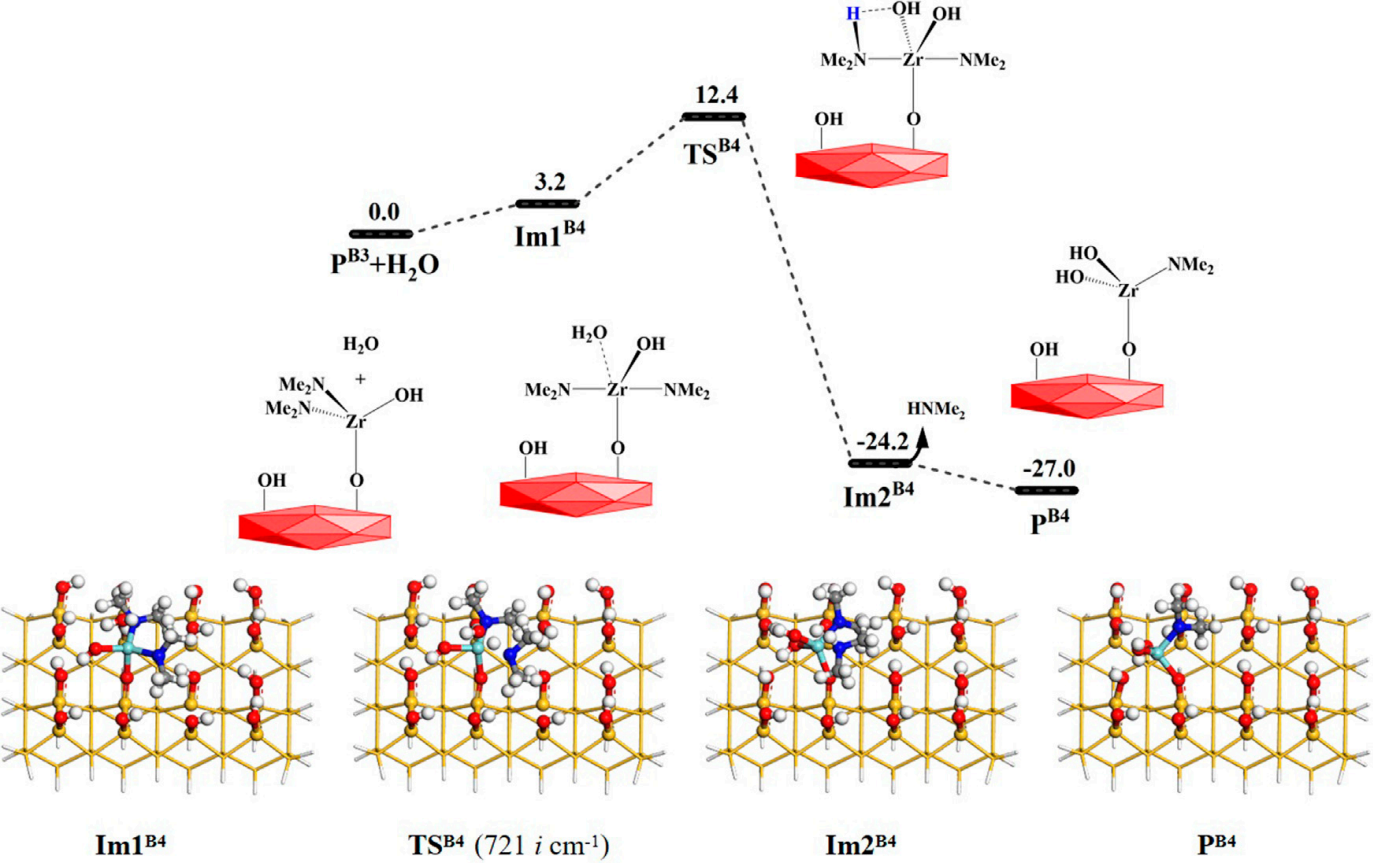
Gibbs free energy (Δ*G*, kcal/mol) profiles of the **B4** section of the H_2_O reaction with the aminated surface.

**FIGURE 9:**
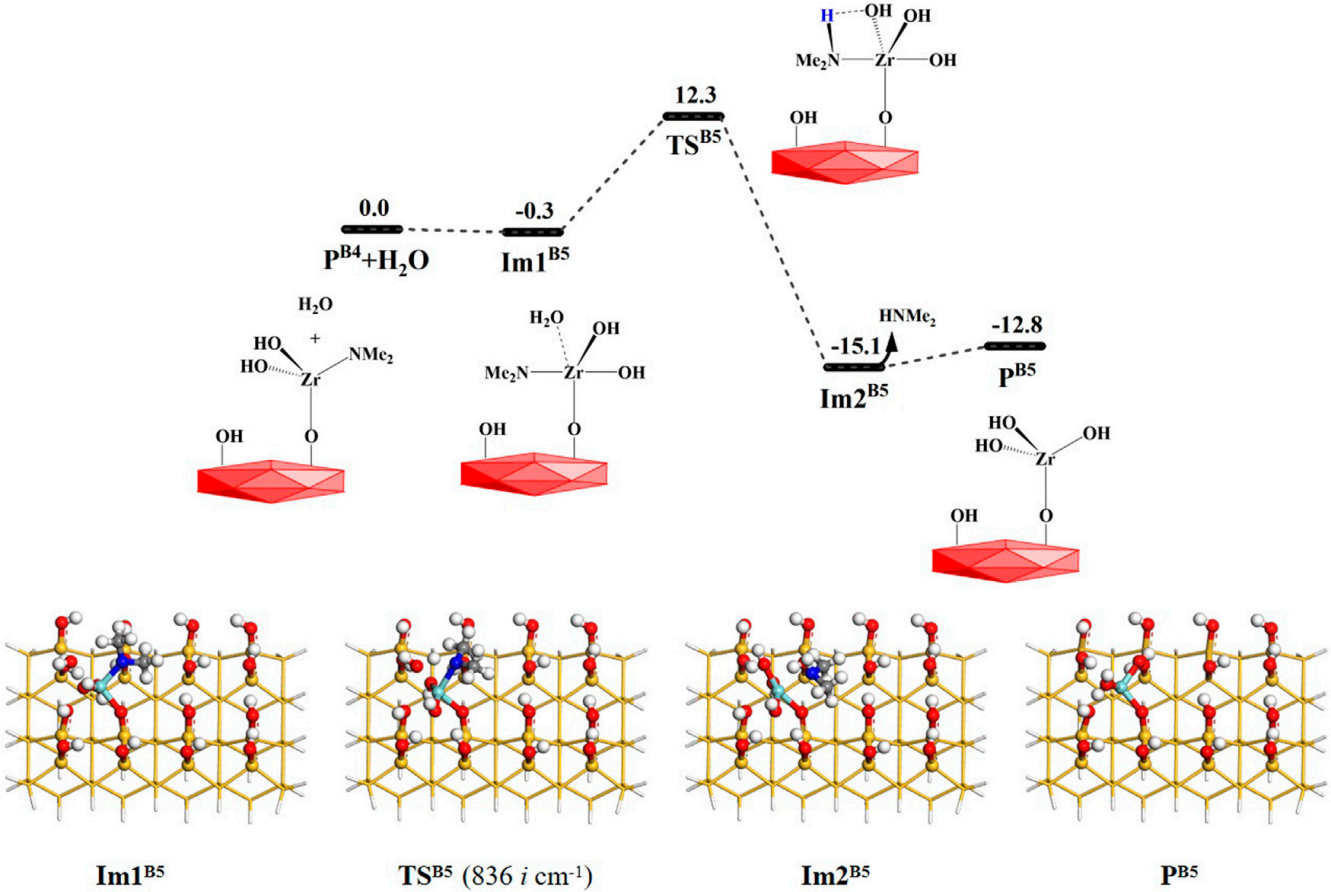
Gibbs free energy (Δ*G*, kcal/mol) profiles of the **B5** section of the H_2_O reaction with the aminated surface.

Similarly, other water molecules can further attack the Zr atom and react with the aminated surface. As shown in Figure 1, the reaction processes of the **B4** and **B5** pathways are similar to that of the **B3** reaction. They can form the intermediates **Im2** ^**B4**^ and **Im2** ^**B5**^ and undergo 4MR transition states **TS** ^**B4**^ and **TS** ^**B5**^, respectively. Lastly, HNMe_2_ can be released and the products **P** ^**B4**^ and **P** ^**B5**^ can be obtained, as shown in Figures 8, 9. The **B4** and **B5** reactions are exoergic by 27.0 and 12.8 kcal/mol and the corresponding Gibbs activation energies are 12.4 and 12.3 kcal/mol, respectively. However, different from the desorption of HNMe_2_ in the **B4** reaction, the release of HNMe_2_ in the **B5** reaction requires a low energy of 2.3 kcal/mol. Supplementary Tables S4, S5 list the changes in the bond lengths at the reaction centers, which indicate that the H–O and Zr–N bonds are cleaved and the H–N and O–Zr bonds are formed.

#### 3.2.3 Coupling reactions between surface amino and hydroxyl groups *via* the B6 and B7 reactions

**FIGURE 10:**
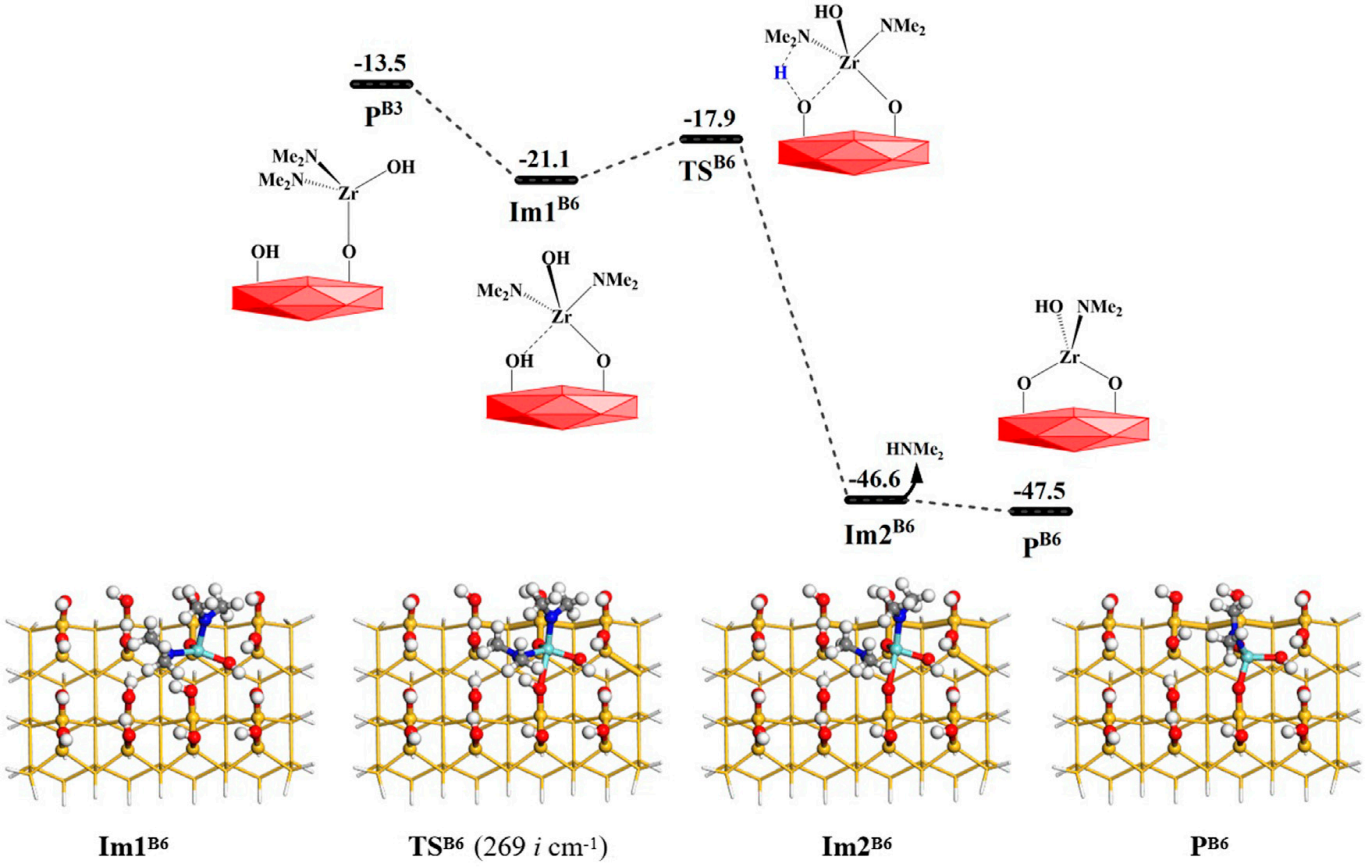
Gibbs free energy (Δ*G*, kcal/mol) profiles of the **B6** section of the H_2_O reaction with the aminated surface.

From Figure 1, it can be seen that amino ligands of the intermediate products **P** ^**B3**^ and **P** ^**B4**^ can react not only with water but also with the adjacent hydroxyl groups on the surface to eliminate the amino ligands and form dimethylamine and bridged products, namely, the coupling reactions between surface amino hydroxyl groups. As shown in Figure 10, the Zr atom of the product **P** ^**B3**^ (–Zr(NMe_2_)_2_OH) can attack the O atom on the adjacent hydroxyl group to form an intermediate **Im1** ^**B6**^ with lower energy. Then, the H atom of the adjacent hydroxyl group on the surface can be transferred to the N atom of the amino ligand to form dimethylamine. It can go through a lower-energy 4MR transition state **TS** ^**B6**^ with the Gibbs activation energy of 3.2 kcal/mol and an intermediate **Im2** ^**B6**^. Finally, HNMe_2_ is released and the intermediate product **P** ^**B6**^ is generated. As a whole, the **B6** reaction is exoergic by 34.0 kcal/mol. As listed in Supplementary Table S6, the lengths of the H–O and Zr–N bonds gradually increase and the lengths of the H–N and O–Zr bonds gradually decrease in the reaction center. The bond lengths of Zr–N and O–H increase from 2.127 and 0.976 Å in **Im1** ^**B6**^ to 2.465 and 2.898 Å in **Im2** ^**B6**^, and the bond lengths of N–H and Zr–O decrease from 2.012 and 2.478 Å to 1.020 and 1.987 Å, respectively.

**FIGURE 11:**
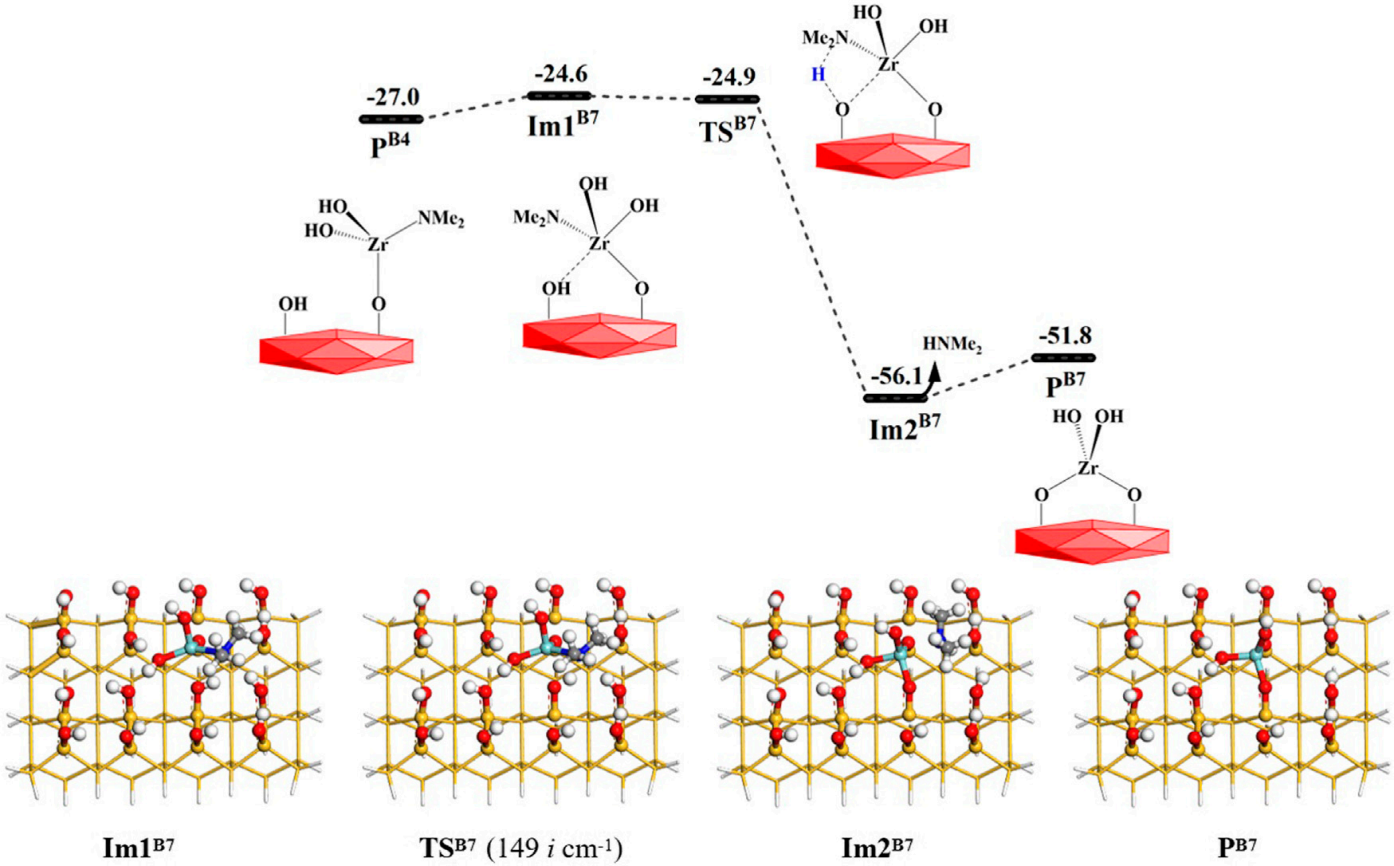
Gibbs free energy (Δ*G*, kcal/mol) profiles of the **B7** section of the H_2_O reaction with the aminated surface.

Similar to the **B6** reaction, the Zr atom of the intermediate product **P** ^**B4**^ (–OZr(NMe_2_)(OH)_2_) can attack the O atom on the surrounding hydroxyl group during the **B7** reaction. It can go through an intermediate **Im1** ^**B7**^ and a hydrogen-transfer transition state **TS** ^**B7**^, in which the H atom on the hydroxyl group can be transferred to the N atom of the dimethylamino group. Lastly, the HNMe_2_ molecule can be released and intermediate product **P** ^**B7**^ (–OZr(OH)_2_O–) can be formed. The **B7** reaction is exoergic by 24.8 kcal/mol and requires a very low Gibbs activation energy of 2.4 kcal/mol. From Figure 11, it can be seen that the Gibbs free energy of intermediate **Im1** ^**B7**^ is higher than that of **TS** ^**B7**^, but the electronic energy of **Im1** ^**B7**^ is lower than that of **TS** ^**B7**^, which results from the overestimation of the thermodynamic correction of **Im1** ^**B7**^ by the harmonic frequency. According to the data in Supplementary Table S7, the tendencies of the breakage of H–O and Zr–N bonds and the formation of H–N and O–Zr bonds are also shown.

#### 3.2.4 Coupling reactions between surface hydroxyl groups *via* the B8 to B10 reactions

In addition, the hydroxyl groups on the intermediate products **P** ^**B3**^, **P** ^**B4**^ and **P** ^**B5**^ can also combine with the adjacent hydroxyl groups to form water molecules, namely the coupling reactions between surface hydroxyl groups. These coupling reactions correspond to **B8**, **B9** and **B10** reactions, shown in Figure 1. The structure of the Zr reaction center can change from a tetrahedral structure to a bridged structure.

**FIGURE 12:**
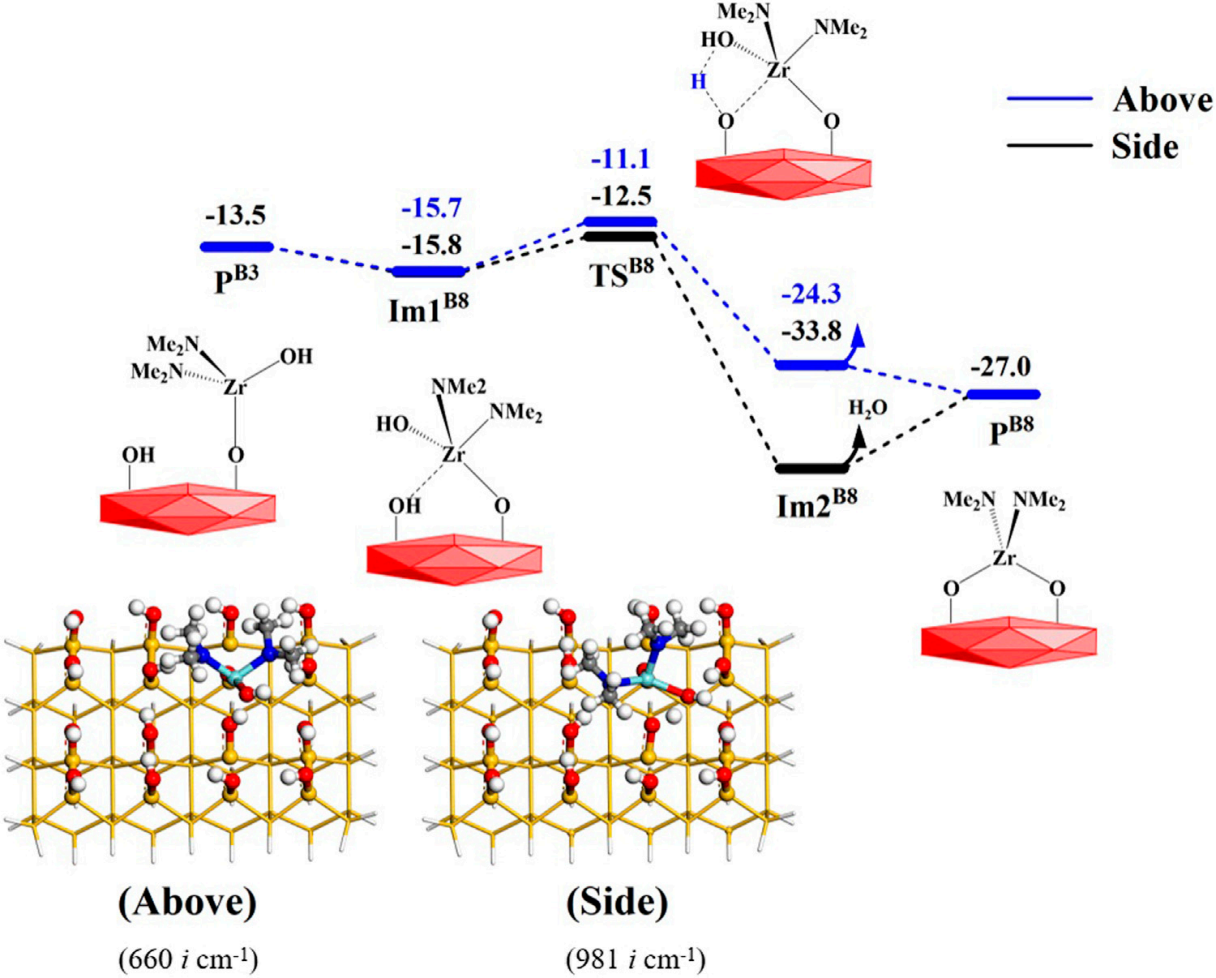
Gibbs free energy (Δ*G*, kcal/mol) profiles of the **B8** section of the H_2_O reaction with the aminated surface.

In the **B8** reaction, the Zr atom of **P** ^**A3**^ can attack the O atom of the adjacent hydroxyl group on the surface to form the intermediate **Im1** ^**B8**^, which is similar to the **B6** reaction. Subsequently, the H atom of the hydroxyl group on the surface can react with the adjacent hydroxyl group to form a water molecule. Considering that the steric hindrance of the hydroxyl group is smaller than that of the dimethylamino group, the hydroxyl group on the Zr atom can react with the adjacent hydroxyl group not only from above but also from the side, as shown in Figure 12. The corresponding Gibbs activation energy is 4.6 or 3.3 kcal/mol, respectively. As a whole, the **B8** reaction is exoergic by 13.5 kcal/mol. As listed in Supplementary Table S8, the changes in the bond lengths of the reaction center show the cleavage of old O–H and Zr–O bonds and the formation of new O–H and Zr–O bonds.

**FIGURE 13:**
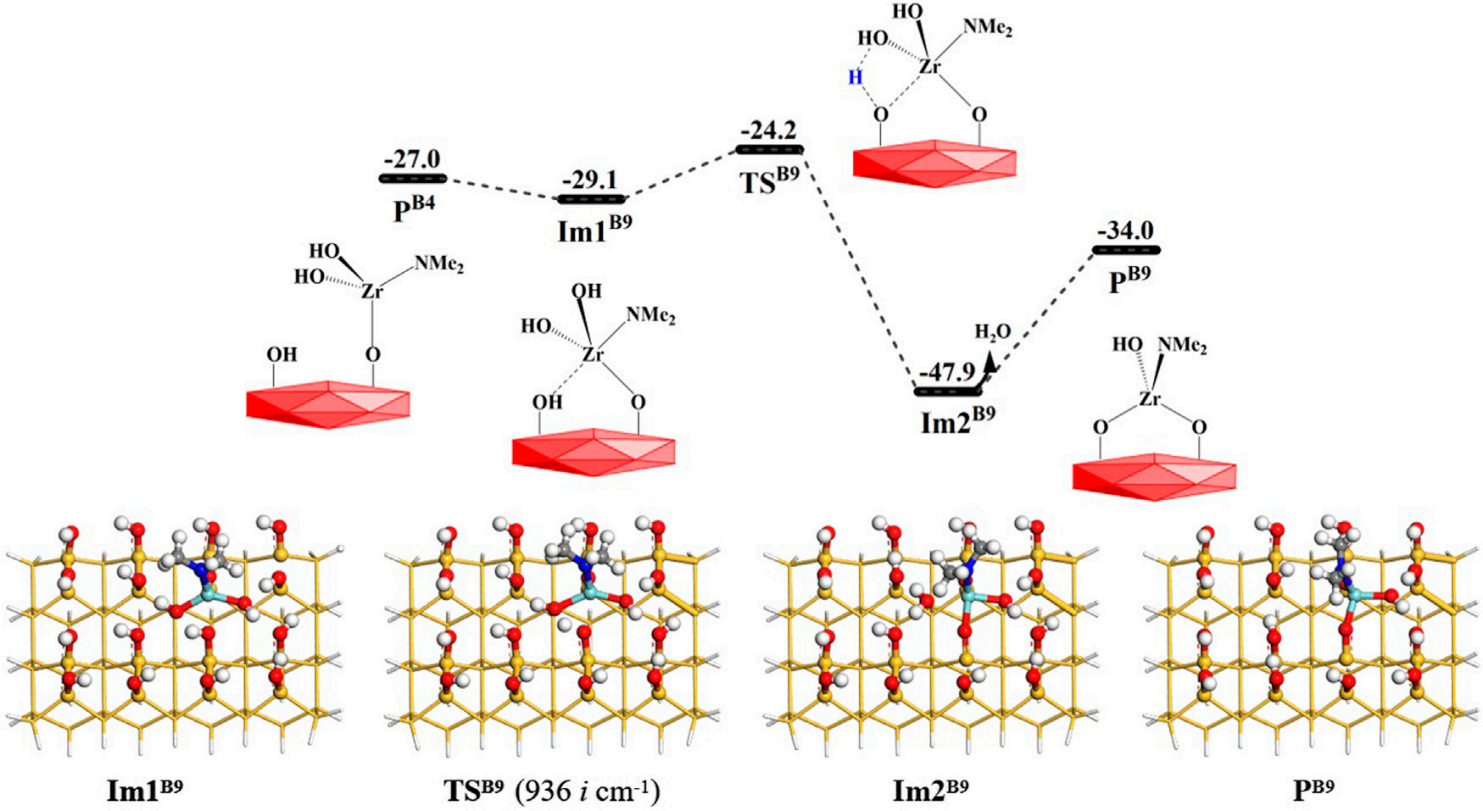
Gibbs free energy (Δ*G*, kcal/mol) profiles of the **B9** section of the H_2_O reaction with the aminated surface.

As shown in Figure 13, the **B9** reaction process is similar to the **B8** section. The Zr atom of intermediate **P** ^**B4**^ can attack the adjacent hydroxyl group to form intermediate **Im** ^**B9**^. Subsequently, the hydroxyl group on the Zr atom can react with the adjacent hydroxyl group through the transition state **TS** ^**B9**^ and the intermediate **Im2** ^**B9**^. Finally, the product **P** ^**B8**^ (–OZr(NMe_2_)(OH)O–) can be obtained. The Gibbs free energy activation energy is about 5.0 kcal/mol. The desorption energy of H_2_O release is about 14.0 kcal/mol. The whole **B9** process is exoergic by 7.0 kcal/mol. From Supplementary Table S9, it can be seen that with the elimination of the hydroxyl group, the lengths of Zr–O′ and O–H bonds increase from 2.004 and 0.970 Å to 2.284 and 2.882 Å, and the lengths of Zr–O and O′–H bonds decrease from 2.398 and 2.491 Å to 2.030 and 0.980 Å, respectively. All these indicate that the Zr–O bond is formed and the –OH group is eliminated.

**FIGURE 14:**
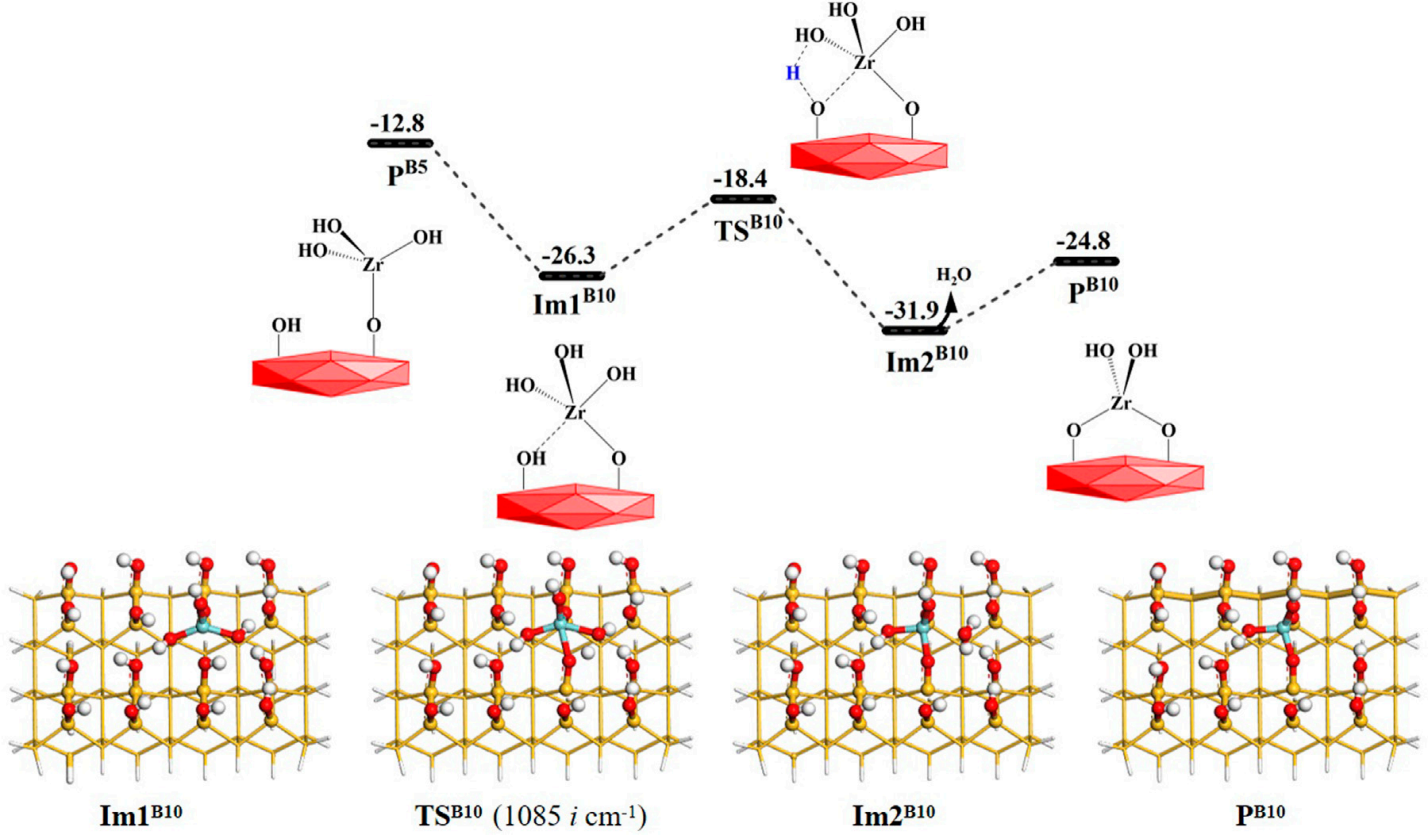
Gibbs free energy (Δ*G*, kcal/mol) profiles of the **B10** section of the H_2_O reaction with the aminated surface.

As shown in Figure 14, the intermediate product **P** ^**B5**^ (–OZr(OH)_3_) of the **B5** reaction can also eliminate a hydroxyl group on the Zr atom by bridged reaction **B10**. The hydroxyl group on **P** ^**B5**^ can react with the H atom on the adjacent hydroxyl group *via* the intermediate **Im** ^**B10**^ and the 4MR transition state **TS** ^**B10**^ to release H_2_O molecules and form the final product **P** ^**B10**^ (–OZr(OH)_2_O). The Gibbs activation energy in the **B10** reaction is 7.9 kcal/mol and the desorption energy of the water molecule is 7.1 kcal/mol. Supplementary Table S10 lists the bond lengths at the reaction center, which indicates the breakage of old bonds and the formation of new bonds in the **B10** section. In comparison with **B6**–**B10**, the Gibbs activation energies of the dimethylamino elimination reactions are lower than those of hydroxyl elimination reactions, which indicates that the elimination reactions of dimethylamine occur relatively easily and are kinetically more favorable.

## 4 Conclusion

Through DFT calculations, possible pathways for the ZrO_2_ ALD reaction of tetrakis(dimethylammonium)zirconium and water on the hydroxylated Si(100) surface were investigated in detail. The whole reaction mechanism includes two main reactions: TDMAZ reactions with the hydroxylated surface and water reactions with the aminated surface. In the TDMAZ reaction, the precursor can eliminate the dimethylamino group by a substitution reaction with the hydroxyl group on the surface. At the same time, the second dimethylamino group of the precursor can be eliminated with the help of other hydroxyl groups on the surface. Considering the configuration of the hydroxylated surface and the Zr–O bond length, only up to two dimethylamines can be eliminated on the Si surface, and the remaining dimethylamine needs to be eliminated by the H_2_O reaction. With increasing temperature, the release of a small molecule adsorbed on the surface takes place more readily. In the H_2_O reaction, the ligand exchange reactions and coupling reactions can alternately occur. In the ligand exchange reactions between the hydroxyl and amino groups, the Gibbs activation energies of the reaction are about 12 kcal/mol, which are the highest in the H_2_O reaction. In the coupling reactions, the hydroxyl or amino groups can react with the neighboring hydroxyl group with lower Gibbs activation energy. Moreover, the coupling reaction of the dimethylamino ligand with the hydroxyl group on the surface is easier than that between the hydroxyl groups on the surface. All these insights into ZrO_2_ ALD could guide the design of new precursors and ALD preparation of other oxides and zirconium compounds.

## Data Availability

The original contributions presented in the study are included in the article/Supplementary Material, further inquiries can be directed to the corresponding authors.
