# Corrigendum: Reaction mechanism of atomic layer deposition of zirconium oxide using zirconium precursors bearing amino ligands and water

**DOI:** 10.3389/fchem.2022.1118819

**Published:** 2022-12-20

**Authors:** Rui Xu, Zhongchao Zhou, Jing Li, Xu Zhang, Yuanyuan Zhu, Hongping Xiao, Lina Xu, Yihong Ding, Aidong Li, Guoyong Fang

**Affiliations:** ^1^ Key Laboratory of Carbon Materials of Zhejiang Province, College of Chemistry and Materials Engineering, Wenzhou University, Wenzhou, China; ^2^ National Laboratory of Solid State Microstructures, College of Engineering and Applied Sciences, Nanjing University, Nanjing, China

**Keywords:** zirconium oxide, atomic layer deposition, reaction mechanism, tetrakis(dimethylamino)zirconium, density functional theory

In the original article, the **Supplementary material** contained “Data Sheet 1”, which was not intended for publication. The incorrect file has been unpublished, and the remaining file, previously “Data Sheet 2”, has been renamed as “Data Sheet 1”.

The authors apologize for this error and state that this does not change the scientific conclusions of the article in any way. The original article has been updated.

