# Supplementary material for: Reaction mechanism of atomic layer deposition of zirconium oxide using zirconium precursors bearing amino ligands and water
Source: Front Chem. 2022 Nov 4;10:1035902. doi: 10.3389/fchem.2022.1035902 (PMC9672480; doi:10.3389/fchem.2022.1035902)
Supplement: Supplementary file 1 [file DataSheet1.PDF]

Supplementary Material

Reaction mechanism of atomic layer deposition of zirconium oxide using zirconium precursors bearing amino ligands and water

Rui Xu[1], Zhongchao Zhou[1], Jing Li[1], Xu Zhang[1], Yuanyuan Zhu[1], Hongping Xiao[1*], Aidong Li[2], Guoyong Fang[1*], Yihong Ding[1], Lina Xu[1*]

[1]Key Laboratory of Carbon Materials of Zhejiang Province, College of Chemistry and Materials Engineering, Wenzhou University, Wenzhou 325035, China

[2]National Laboratory of Solid State Microstructures, College of Engineering and Applied Sciences, Nanjing University, Nanjing 210093, China

***Correspondence:**

Hongping Xiao, Guoyong Fang and Lina Xu

hp_xiao@wzu.edu.cn, fanggy@wzu.edu.cn and xulina@wzu.edu.cn

Computations of the oxidation state of Zr element

In order to study the change of the oxidation state of Zr atom in the reaction process, the atomic orbitals of the structures at all stationary points except the transition states were localized using Multiwfn 3.8 program [S1]. By the localized orbital bonding analysis (LOBA) method, the oxidation state of Zr atom was analyzed [S2]. The results show that the oxidation state of Zr atom is four and does not change in the reactants, intermediates and products during the whole reaction.

References:

[S1] T. Lu, F. Chen, Multiwfn: a multifunctional wavefunction analyzer, J. Comput. Chem. 33 (2012) 580-592.

[S2] A.J.W. Thom, E.J. Sundstrom, M. Head-Gordon, LOBA: a localized orbital bonding analysis to calculate oxidation states, with application to a model water oxidation catalyst, Phys. Chem. Chem. Phys. 11 (2009) 11297-11304.
